# Evaluation of Sarcopenia in Patients with Monoclonal Gammopathy of Undetermined Significance

**DOI:** 10.3390/jcm13123458

**Published:** 2024-06-13

**Authors:** Ayse Nilgun Kul, Mujgan Kaya Tuna

**Affiliations:** 1 Department of Hematology, Prof. Dr. Ilhan Varank City Hospital, Istanbul 34785, Turkey; 2 Department of Family Medicine and Obesity, Kartal Dr. Lutfi Kırdar City Hospital, Istanbul 34865, Turkey; mjgn-tn@hotmail.com

**Keywords:** MGUS, sarcopenia, prevalence, muscle mass, multiple myeloma

## Abstract

**Background:** We aimed to determine the prevalence of sarcopenia in patients with monoclonal gammopathy of undetermined significance (MGUS) and to evaluate the links between MGUS and sarcopenia. **Methods:** Eighty-two patients with a diagnosis of MGUS were enrolled in the study. Muscle strength was measured using the handgrip dynamometer. Physical performance was assessed by assessing gait speed over a 6-minute walking test. Muscle mass was determined using a bioelectrical impedance analyzer. **Results:** Sarcopenia was confirmed in 34.15% of patients. Male predominance was demonstrated in MGUS subjects with sarcopenia, particularly patients with low hand grip strength, low appendicular skeletal muscle mass (ASMM), or low ASMM index (*p* < 0.001, 0.013, and 0.001, respectively). Higher age and lower serum free light-chain Lambda levels were shown in MGUS patients with low muscle function scores compared to normal scores (*p* < 0.001, and 0.014, respectively). In addition, having a low ASMM score was related to low body mass index and high-risk group (*p* = 0.020, 0.033, respectively). **Conclusions:** We demonstrated that the frequency of sarcopenia is high in patients with MGUS. Whether sarcopenia has a possible role as a factor contributing to the pathogenesis of MGUS should be supported by further studies containing longitudinal data.

## 1. Introduction

Monoclonal gammopathy of undetermined significance (MGUS) is an asymptomatic plasma cell dyscrasia that occurs in approximately 3% of the population over the age of 50, and its prevalence increases with age [1]. MGUS is characterized by serum monoclonal protein, but with levels less than 3 g/dL. Other findings include plasma cell infiltration in the bone marrow (<10%) and lack of evidence for end-organ damage, especially with the exclusion of chronic renal disease, anemia, hypercalcemia, and lytic bone lesions [2]. It is perceived as a premalignant condition that is estimated to have an annual likelihood of 1% to progress into multiple myeloma or lymphoplasmacytic malignancies [3]. Malignant progression has been associated with abnormal serum free light chain (FLC) levels, the presence of non-IgG MGUS, and a relatively higher monoclonal protein in the serum (≥1.5 g/dL). Multiple risk factors appear to have an exponential impact on malignancy development, where one, two, and three risk factors, respectively, lead to a 5%, 21%, and 58% risk for malignancy [3]. Regardless of its malignant potential, MGUS may have adverse effects on the cardiovascular system, renal system, and bone metabolism, increasing the risks for cardiovascular events, renal failure, and fractures [4]. Identifying other factors associated with pathophysiology or progression may provide data to improve monitoring, clinical decision-making, and management, as well as elucidate the underlying pathogenesis of monoclonal gammopathies.

Sarcopenia is a muscular condition marked by a widespread and often gradual decline in muscle strength and mass, which diminishes physical capabilities. It has various detrimental consequences, including physical disability, fracture, reduced independence, poor quality of life, and increased mortality [5]. Despite the fact that it can rarely occur earlier in life, it is predominantly an age-dependent issue which is in parallel with MGUS [6]. Sarcopenia prevalence ranges from 10% to 27% in the elderly population, and its risk factors are age, sex, malnutrition, sedentary lifestyle, chronic diseases, and cancers [7]. These factors are also common among patients with MGUS [8]. MGUS outcomes may also predispose to muscle weakness and/or loss of physical function, whereas muscle atrophy itself can result in adverse immune outcomes [8,9,10]. Despite these bidirectional relationships between sarcopenia and MGUS, there is a lack of data in the literature on the prevalence of sarcopenia in patients with MGUS and the risk factors that may lead to its development.

Our aim was to determine the frequency of sarcopenia in Turkish patients with MGUS and to evaluate the potential relationships between MGUS and sarcopenia-associated factors, such as muscle strength, muscle function, and muscle quantity or quality.

## 2. Materials and Methods

### 2.1. Study Design and Setting

The study was approved by the Ethics Committee of Dr. Lütfi Kırdar Kartal City Hospital (Decision date: 8 February 2023, decision no. 2023/51,1/2,13/l) and was carried out in accordance with the ethical standards stated in the Declaration of Helsinki and its amendments. Informed consent was obtained from all subjects involved in the study. This was a single-center, cross-sectional study which was conducted between February 2023 and August 2023 in the Hematology outpatient clinic of Lütfi Kırdar Kartal City Hospital, Istanbul, Turkey. A total of 82 patients who had a diagnosis of MGUS were enrolled in the study. The diagnosis of MGUS in all patients had been made based on the diagnostic criteria of the International Myeloma Working Group (2010). As such, the following criteria had been utilized at the time of diagnosis to define MGUS: (i) the presence of <3 g/dL monoclonal protein (M protein) in serum; (ii) <10% monoclonal plasma cells in the bone marrow; and (iii) the absence of end organ damage, such as hypercalcemia, renal failure, anemia, or bone lesions [2]. In addition, we confirmed that patients did not have light chain amyloidosis and did not meet the diagnostic criteria for lymphoma or lymphoplasmacytic malignancies.

Participants younger than 18 years or older than 85 years; those with a history of muscle disease, neuropathy, secondary malignancy, severe psychiatric disorders such as bipolar disorder or psychosis, and malnutrition; and those receiving chronic corticosteroid therapy were excluded from the study. Furthermore, we excluded from the study patients with abnormal electrolyte, thyroid stimulating hormone, and creatine kinase measurements.

### 2.2. Disease Classification

Based on the Mayo Clinic’s stratification model, patients with MGUS were classified as high, high–intermediate, low–intermediate, and low risk according to three factors: abnormal serum FLC ratio, non-IgG MGUS presence, and relatively high serum M protein level (≥1.5 g/dL) [3]. In the presence of all three factors, the patient was defined to have high-risk MGUS. If any two were present, the definition was high-intermediate-risk MGUS. A single risk factor was accepted to show low-intermediate-risk MGUS. Finally, patients without any of these factors were defined as having low-risk MGUS. The high and high-intermediate risk groups were pooled to create the high-risk group examined in this study. Also, the low and low–intermediate groups were pooled to create the low-risk group.

### 2.3. Examined Data

Demographic and clinical features, including age, sex, body mass index (BMI), and comorbidities, were obtained from medical files. Body weight (in kilograms) was measured while subjects wore light clothing, using a calibrated balance beam scale. Height was measured using a stadiometer. BMI was calculated as weight divided by height squared (kg/m^2^). All measurements and evaluations were performed by the same trained researchers to avoid inter-observer variability as much as possible.

### 2.4. Sarcopenia Evaluation

All patients were evaluated for sarcopenia. The diagnosis of sarcopenia was based on the revised criteria of European Working Group on Sarcopenia in Older People (EWGSOP2—2018), which recommends three main factors to consider: low muscle strength, low muscle quantity or quality, and low physical performance [11]. The handgrip strength (HGS) test was used for muscle strength assessment, which was quantified using a strain-gauged dynamometer (Takei TKK 5001 model, Takei Scientific Instruments Co. Ltd., Tokyo, Japan). During measurements, the subject was in a standing position with his/her arms parallel to the body but without contact with the torso. Participants were asked to perform two maximum-force trials with each hand, and the maximum value was recorded. Using the cut-off values proposed in the EWGSOP2 Consensus, low HGS was defined as <27 kg for males and <16 kg for females. The presence of low HGS was defined as low muscle strength. Muscle mass was measured using a bioelectrical impedance analyzer (Tanita Body Composition Analyzer MC-580 model, Tanita Co., Tokyo, Japan). The estimated appendicular skeletal muscle mass (ASMM) was determined by the Sergi equation, and an index value was determined by adjusting for the body surface (ASMM/height^2^). Applying specific cut-off points recommended by EWGSOP2, sarcopenia was defined as ASMM < 20 kg in males and ASMM < 15 kg in females. Low muscle quantity was identified as ASMM index < 7.0 kg/m^2^ in males and <5.5 kg/m^2^ in females. Physical performance was evaluated using the usual gait speed, using the 6-minute walking test (6MWT). Participants were instructed to walk at their usual pace, covering as much distance as possible without interruption along a lengthy corridor featuring flat, hard, and smooth flooring, over a duration of 6 min. Walking speed was computed by dividing the distance in meters by the time in seconds for each participant, resulting in a meters per second value (m/s). A gait speed below the cut-off value of <0.8 m/s was defined as a low gait speed. Additionally, patients who walked less than 400 m were defined as having a low gait speed.

### 2.5. Biochemical Analysis

Blood samples were drawn from the antecubital vein after 12 h of fasting on the day of hospital admission and were centrifuged at 5000 rpm (1500× *g*) for 10 min to separate the serum. M protein level was determined using serum protein electrophoresis. Serum IgG, IgA, and IgM were measured via the nephelometric method on a Beckman IMMAGE system (Beckman Coulter, Indianapolis, IN, USA). Serum-FLC (sFLC) Kappa and Lambda levels were determined by the monoclonal antibody-based nephelometric method on a Siemens BN system (Siemens Healthcare Diagnostics GmbH, Marburg, Germany), and FLC ratios were calculated.

### 2.6. Statistical Analysis

The data obtained throughout the study were recorded and analyzed using SPSS version 25.0 software (IBM Corporation, Armonk, New York, NY, USA). The normality of the numerical data distribution was assessed using histogram and Q-Q plots. Numerical variables with normal distribution were presented as mean and standard deviation and were compared between groups, using Student’s *t*-test. Numerical variables which were non-normally distributed were presented with median and 25th–75th percentiles and were compared between groups, using the non-parametric Mann-Whitney U test. Categorical variables were expressed as count (*n*) with percentages and were compared using chi-square tests or the Fisher’s exact test. A *p*-value of <0.05 was considered statistically significant.

## 3. Results

Eighty-two patients diagnosed with MGUS were enrolled in the study. Demographic and clinical characteristics are summarized in Table 1. The mean age of patients was 62.44 ± 10.95 years, and 43 patients (52.44%) were female. The patients had a mean BMI of 30.28 ± 5.98 kg/m^2^. Hypertension (*n*:38, 46.34%) and diabetes mellitus (*n*:25, 30.49%) were the most common comorbidities. While the median M protein level was 4 (2.1–6.7) g/dL, the number of patients with a level of <3 g/dL was 29 (35.37%). Regarding the types of MGUS, we detected IgA, IgG, and IgM MGUS in 15.85%, 69.51%, and 14.63% of cases, respectively. The median level of the sFLC Kappa-to-Lambda ratio was 1.078 (0.739–1.693), and 23 (28.05%) patients had an sFLC ratio of ≥1.65. Thirty-eight (46.34%) patients were categorized as high-risk MGUS.

Evaluation of patients with regard to sarcopenia diagnosis criteria are reported in Table 2. Sarcopenia was confirmed in 34.15% (*n*:28) of all patients. The mean grip strength was computed as 22.35 ± 5.57 kg, and 40 patients showed low muscle strength. While the mean ASMM was found to be 19.65 ± 4.14, low ASMM was observed in 24 (29.27%) patients. The 6MWT was completed by all patients, and the median value was 374 (289–425) meters. Forty-six (56.1%) patients presented with low gait speed. The mean ASMM index value was 7.19 ± 1.38 kg/m^2^, and 23 patients were categorized as having low ASMM.

The study population was divided into two groups according to the presence of sarcopenia (Table 3). MGUS patients with sarcopenia had a higher proportion of males (*p* = 0.001). In the low-risk MGUS group, 32 (72.73) patients did not meet the criteria for sarcopenia, while 12 (27.27%) exhibited sarcopenia. In the high-risk MGUS group, confirmed sarcopenia was observed in 16 (42.11%) subjects (*p* = 0.238). No significant differences were observed between groups in terms of age, comorbidities, BMI value, M protein level, and sFLC ratio (all, *p* > 0.05).

MGUS patients were categorized into subgroups with regard to the diagnostic criteria of sarcopenia. Higher male frequencies were found in MGUS patients with low hand grip strength, low ASMM, or low ASMM index (*p* < 0.001, *p* = 0.013, and *p* = 0.001, respectively) (Table 4, Table 5 and Table 6). BMI was lower in MGUS patients with low ASMM (27.90 ± 5.81 kg/m^2^) than in normal ASMM patients (31.26 ± 5.81 kg/m^2^) (*p* = 0.020) (Table 5). Eight (33.33%) patients in the low-risk group had low ASMM, while sixteen (66.67%) patients in the high-risk group had low ASMM (*p* = 0.033) (Table 5). MGUS patients with low performance scores were older compared to those with normal scores (*p* < 0.001) (Table 7). sFLC Lambda levels were found to be 32.70 (21.15–50.65) mg/L among patients with normal gait speed and 48.85 (29.70–79.70) mg/L among those with low gait speed (*p* = 0.014) (Table 7).

## 4. Discussion

The aim of the study was to assess the prevalence of sarcopenia and its potential relationships with MGUS. Nearly one-third of MGUS patients exhibited confirmed sarcopenia. Among MGUS subjects with sarcopenia, a male predominance was evident, particularly in those displaying low hand grip strength and low ASMM (both absolute and adjusted index). Despite lack of statistical significance, high-risk MGUS appeared to be somewhat more common in patients with sarcopenia. Furthermore, MGUS patients with diminished muscle function scores were older and had lower levels of sFLC Lambda compared to those with normal scores. Additionally, low ASMM scores were associated with lower BMI and high-risk MGUS. These findings reveal that sarcopenia should be considered among MGUS patients, irrespective of severity. It is worth noting that comorbidities were prevalent in our patient cohort, which is expected given the advanced age of the group. However, this high prevalence of comorbidities may have obscured potential associations between MGUS and sarcopenia.

The skeletal muscle is a complex, dynamic, and secretory organ comprising approximately 40–50% of body mass, which is critically involved in metabolic homeostasis [12]. It releases bioactive molecules—referred to as myokines—that exert endocrine, paracrine, and autocrine functions [13]. Impaired muscle function and loss of skeletal muscle mass are key features of cancer cachexia and sarcopenia. In addition, some myokines exhibit antineoplastic effects, yielding an essential role for sarcopenia in oncology. Factors causing sarcopenia in neoplastic patients can be mainly attributed to reduced food intake, anorexia, side effects of therapies, and muscle disuse. In fact, numerous studies reveal a complex interplay between energy balance, immune regulation, inflammatory response, glucose metabolism, and body muscle mass [14,15,16]. Sarcopenia has been related to a worse prognosis and the inability to tolerate optimal management in neoplastic patients, resulting in treatment-related toxicity and other complications [17]. In addition, cancer therapies have negative effects on body composition, primarily by leading cachexia and muscle wasting, as well as loss of fat tissue and bone mass [18]. The prevalence of sarcopenia varies depending on age group, sex, ethnicity, and, perhaps most crucially, the diagnostic criteria. Comprehensive studies have estimated frequencies of 10% in the general elderly population, 14.7% in hospitalized older patients, and 38.6% in cancer patients [19]. The prevalence of sarcopenia in the Turkish population was 11.8% among elderly individuals living in rural areas and 21.6% in those living in urban centers [20]. A meta-analysis of 1578 patients with different hematological malignancies showed that sarcopenia was diagnosed in 39.1% of patients with relatively low survival [9]. Recently, Zeng et al. revealed in a systemic review of 3354 patients with malignant hematological malignancies that sarcopenia had a prevalence of over 30% in patients with a poorer prognosis, and that older subjects presented with a higher sarcopenic rate compared to younger individuals [21]. Consistent with the literature, we demonstrated a high frequency of sarcopenia in MGUS patients, with 34.15% confirmed cases. Despite not reaching statistical significance, high-risk MGUS was relatively more common among patients with sarcopenia, which is an important potential link that must be examined in future studies that include a larger number of patients. Taken together with the literature, our data support the notion that sarcopenia and MGUS can interact with and aggravate each other, and sarcopenia may contribute to clinical adverse consequences of MGUS by decreasing quality of life and limiting functionality and/or independence. Based on the known characteristics and pathological background of sarcopenia (mentioned above), it appears that the possible mechanisms in this relationship may be based on mitochondrial dysfunction, metabolic dysfunctions, and increased catabolism. A periodic assessment of muscle mass, strength, and performance may allow for the diagnosis of sarcopenia in early stages in order to facilitate well-planned intervention to limit the detrimental impact of sarcopenia among patients with MGUS. These possibilities could contribute to the improvement of MGUS management.

Muscle diseases can be misdiagnosed or overlooked because they are relatively rare and sometimes may not present with specific symptoms. There are a limited number of studies in the literature investigating myopathies associated with monoclonal gammopathies. Yu et al. published a case report concerning a 60-year-old male with symmetric proximal limb fatigue and muscle weakness, with the most particular highlight being the demonstration of the importance of comprehensive screening for monoclonal gammopathies of clinical significance (MGCS)-associated myopathy [22]. Although the general consensus is that MGUS causes myopathy through monoclonal immunoglobulins and their impact on sarcomeric proteins of myocytes, the existing literature suggests that MGUS patients may be predisposed to muscle diseases through unclear and complex mechanisms. It is therefore possible that future research examining relationships between MGUS and sarcopenia may reveal other mechanisms that lead to muscle loss or functional impairment.

Sex differences have been reported rather frequently in both MGUS and sarcopenic patients, including adverse outcomes, with higher predisposition among males [23,24]. Consistent with the literature, we demonstrated male predominance in MGUS subjects with sarcopenia, particularly patients with low hand grip strength, low ASMM, and low ASMM index. This may be related to the fact that males tend to demonstrate a greater loss of muscle mass in older age, whereas the decrease in muscle mass is much lesser among females. In addition, with advancing age, males experience a deficit in testosterone and insulin-like growth factor-1 levels which can lead to a rapid loss of muscle mass, function, and strength, thereby increasing the likelihood of sarcopenia in older males.

Although obesity has been reported to be related to multiple myeloma risk, studies on BMI measurements with MGUS are limited and inconclusive. Kleinstern et al. demonstrated in 594 MGUS patients that high BMI was an independent prognostic indicator for MGUS progression, even after adjustment for M protein level, isotype (Ig type), and FLC ratio, particularly among females [25]. In contrast, a case–control study of 100 MGUS patients progressing to multiple myeloma or other malignancies and 100 MGUS controls without progression showed that obesity (defined as BMI > 30) was unassociated with progression [26]. We show that low muscle mass is associated with high BMI in patients with MGUS. Previous studies, including our own, are unable to establish whether BMI is a factor influencing MGUS onset or whether it increases the risk of progression to malignancy. Discrepancies in racial characteristics, cultural backgrounds, dietary habits, and levels of physical activity could be decisive in this matter and may have altered the conclusions of different studies. Therefore, further data are necessary to draw definitive links between BMI and MGUS or its progression.

Patients with MGUS have an annual risk of malignant progression of approximately 1%, and a common risk score to estimate the likelihood of progression has been developed by the Mayo Clinic based on the serological subtype of MGUS, M protein level, and FLC ratio [3]. We implemented this stratification model, identifying 38 patients as high risk. The only notable association with MGUS risk was determined to be low muscle mass, which is a sub-diagnostic criterion of sarcopenia. This finding supports the existing literature. Anabtawi et al. recently conducted a meta-analysis of 20 studies encompassing 3468 patients with various hematological malignancies. They found that the presence of low skeletal muscle mass in individuals with cancer was associated with poor progression-free survival, overall survival, and mortality, regardless of the specific type of malignancy [27]. Our results provide some evidence that associates low muscle mass with adverse survival in MGUS patients. Future well-designed prospective studies are necessary to completely evaluate causality and determine the underlying mechanisms.

### Strengths and Limitations

The cross-sectional design prevents causal interpretations. The data were drawn from a single center, and the relatively small number of participants limits the analyses with respect to the heterogeneity of MGUS subtypes. Our study also lacks data concerning sarcopenia or changes in body composition before the onset of MGUS, which might prove crucial to understanding the directional relationship between MGUS and sarcopenia and could also reveal longitudinal tendencies regarding these parameters. Although the physical activities of the participants in our study were evaluated with a 6MWT, not using a measurement tool such as an accelerometer that evaluates daily physical activities with the dose–response relationship between muscle mass/strength may have contributed to our results [28]. Finally, the lack of exact information regarding the progression of MGUS among our patients has made it impossible to pinpoint the exact impact of sarcopenia on disease-related outcomes. In addition, the literature is very limited in terms of comparable data from studies that assessed potential relationships between MGUS and sarcopenia, thus hindering the interpretation of the current findings relative to other settings.

## 5. Conclusions

To the best of our knowledge, this is one of the very few studies examining the relationship between MGUS and sarcopenia. We demonstrated that the frequency of sarcopenia is high in patients with MGUS. Early identification of sarcopenia in MGUS patients may help identify patients at risk, prevent progression associated with poor outcomes, and improve quality of life. However, more studies focusing on this topic are needed to arrive upon generalizable conclusions.

## Figures and Tables

**Table 1:** Demographics and clinical characteristics of the study population.

Variables	Result
Age	62.44 ± 10.95
Sex	
Female	43 (52.44%)
Male	39 (47.56%)
Comorbidity	
Hypertension	38 (46.34%)
Diabetes mellitus	25 (30.49%)
Ischemic heart disease	18 (21.95%)
Hypothyroidism	6 (7.32%)
COPD	3 (3.66%)
Hyperlipidemia	2 (2.44%)
Body mass index, kg/m^2^	30.28 ± 5.98
M protein level, g/dL	4.00 (2.10–6.70)
<3	29 (35.37%)
≥3	53 (64.63%)
M protein type	
IGG	57 (69.51%)
IGA	13 (15.85%)
IGM	12 (14.63%)
sFLC Kappa, mg/L	46.10 (24.50–82.02)
sFLC Lambda, mg/L	40.05 (27.10–74.90)
sFLC Kappa-to-Lambda ratio	1.078 (0.739–1.693)
<1.65	59 (71.95%)
≥1.65	23 (28.05%)
Risk score	
Low	44 (53.66%)
High	38 (46.34%)

sFLC, serum free light chain; COPD, chronic obstructive pulmonary disease. Descriptive statistics are presented using mean ± standard deviation for normally distributed numerical variables, median (25th percentile–75th percentile) for non-normally distributed numerical variables and count (percentage) for categorical variables.

**Table 2:** Evaluation of patients with regard to sarcopenia diagnosis criteria.

Variables	Result
Grip strength, kg (F/M)	20.45 ± 5.14/24.43 ± 5.33
Normal	42 (51.22%)
Low	40 (48.78%)
ASMM, kg (F/M)	18.54 ± 3.95/20.88 ± 4.04
Normal	58 (70.73%)
Low	24 (29.27%)
ASMM index, kg/m^2^ (F/M)	7.16 ± 1.46/7.22 ± 1.30
Normal	54 (65.85%)
Low	28 (34.15%)
Six-minute walk test, meters	374 (289–425)
Normal	36 (43.90%)
Low	46 (56.10%)
Sarcopenia confirmed	
No	54 (65.85%)
Yes	28 (34.15%)

ASMM, appendicular skeletal muscle mass; F, female; M, male. Descriptive statistics are presented using mean ± standard deviation for normally distributed numerical variables, median (25th percentile–75th percentile) for non-normally distributed numerical variables, and count (percentage) for categorical variables.

**Table 3:** Demographics and clinical characteristics of patients with regard to sarcopenia.

	Sarcopenia		
Variables	No (*n* = 54)	Yes *(n* = 28)	*p*
Age	61.70 ± 10.67	63.86 ± 11.54	0.402 ^†^
Sex			
Female	36 (66.67%)	7 (25.00%)	0.001 ^§^
Male	18 (33.33%)	21 (75.00%)	
Comorbidity			
Hypertension	23 (42.59%)	15 (53.57%)	0.477 ^§^
Diabetes mellitus	17 (31.48%)	8 (28.57%)	0.985 ^§^
Ischemic heart disease	10 (18.52%)	8 (28.57%)	0.446 ^§^
Hypothyroidism	5 (9.26%)	1 (3.57%)	0.659 ^#^
COPD	2 (3.70%)	1 (3.57%)	1.000 ^#^
Hyperlipidemia	2 (3.70%)	0 (0.00%)	0.545 ^#^
Body mass index, kg/m^2^	31.17 ± 5.95	28.56 ± 5.75	0.060 ^†^
M protein level, g/dl	4.00 (2.10–6.90)	3.85 (2.31–6.15)	0.796 ^‡^
<3	21 (38.89%)	8 (28.57%)	0.495 ^§^
≥3	33 (61.11%)	20 (71.43%)	
M protein type			
IGG	39 (72.22%)	18 (64.29%)	0.626 ^§^
Other	15 (27.78%)	10 (35.71%)	
sFLC Kappa, mg/L	47.14 (24.50–89.70)	45.70 (29.19–76.09)	0.868 ^‡^
sFLC Lambda, mg/L	34.35 (26.30–75.00)	50.55 (28.25–71.05)	0.509 ^‡^
sFLC Kappa-to-Lambda ratio	1.114 (0.839–1.929)	0.946 (0.687–1.655)	0.302 ^‡^
<1.65	39 (72.22%)	20 (71.43%)	1.000 ^§^
≥1.65	15 (27.78%)	8 (28.57%)	
Risk score			
Low	32 (59.26%)	12 (42.86%)	0.238 ^§^
High	22 (40.74%)	16 (57.14%)	

COPD, chronic obstructive pulmonary disease. Descriptive statistics are presented using mean ± standard deviation for normally distributed numerical variables, median (25th percentile–75th percentile) for non-normally distributed numerical variables, and count (percentage) for categorical variables. ^†^ Student’s *t*-test; ^‡^ Mann–Whitney U test; ^§^ chi-square test; ^#^ Fisher’s exact test.

**Table 4:** Demographics and clinical characteristics of patients with regard to hand grip strength.

	Hand Grip Strength		
Variables	Normal (*n* = 42)	Low (*n* = 40)	*p*
Age	60.79 ± 10.56	64.17 ± 11.22	0.163 ^†^
Sex			
Female	33 (78.57%)	10 (25.00%)	<0.001 ^§^
Male	9 (21.43%)	30 (75.00%)	
Comorbidity			
Hypertension	16 (38.10%)	22 (55.00%)	0.189 ^§^
Diabetes mellitus	11 (26.19%)	14 (35.00%)	0.531 ^§^
Ischemic heart disease	6 (14.29%)	12 (30.00%)	0.147 ^§^
Hypothyroidism	5 (11.90%)	1 (2.50%)	0.202 ^#^
COPD	2 (4.76%)	1 (2.50%)	1.000 ^#^
Hyperlipidemia	1 (2.38%)	1 (2.50%)	1.000 ^#^
Body mass index, kg/m^2^	31.04 ± 6.42	29.48 ± 5.44	0.239 ^†^
M protein level, g/dL	4.05 (2.10–6.60)	3.80 (2.31–7.10)	0.578 ^‡^
<3	17 (40.48%)	12 (30.00%)	0.447 ^§^
≥3	25 (59.52%)	28 (70.00%)	
M protein type			
IGG	31 (73.81%)	26 (65.00%)	0.531 ^§^
Other	11 (26.19%)	14 (35.00%)	
sFLC Kappa, mg/L	47.14 (22.80–90.67)	45.70 (33.76–81.54)	0.933 ^‡^
sFLC Lambda, mg/L	33.35 (24.10–57.70)	50.55 (30.05–75.10)	0.154 ^‡^
sFLC Kappa-to-Lambda ratio	1.213 (0.839–2.070)	1.014 (0.694–1.531)	0.128 ^‡^
<1.65	28 (66.67%)	31 (77.50%)	0.398 ^§^
≥1.65	14 (33.33%)	9 (22.50%)	
Risk score			
Low	23 (54.76%)	21 (52.50%)	1.000 ^§^
High	19 (45.24%)	19 (47.50%)	

COPD, chronic obstructive pulmonary disease. Descriptive statistics are presented using mean ± standard deviation for normally distributed numerical variables, median (25th percentile–75th percentile) for non-normally distributed numerical variables, and count (percentage) for categorical variables. ^†^ Student’s *t*-test; ^‡^ Mann–Whitney U test; ^§^ chi-square test; ^#^ Fisher’s exact test.

**Table 5:** Demographics and clinical characteristics of the patients with regard to ASMM.

	ASMM		
Variables	Normal (*n* = 58)	Low (*n* = 24)	*p*
Age	61.43 ± 10.84	64.87 ± 11.07	0.197 ^†^
Sex			
Female	36 (62.07%)	7 (29.17%)	0.013 ^§^
Male	22 (37.93%)	17 (70.83%)	
Comorbidity			
Hypertension	27 (46.55%)	11 (45.83%)	1.000 ^§^
Diabetes mellitus	18 (31.03%)	7 (29.17%)	1.000 ^§^
Ischemic heart disease	12 (20.69%)	6 (25.00%)	0.892 ^§^
Hypothyroidism	5 (8.62%)	1 (4.17%)	0.666 ^#^
COPD	1 (1.72%)	2 (8.33%)	0.204 ^#^
Hyperlipidemia	2 (3.45%)	0 (0.00%)	1.000 ^#^
Body mass index, kg/m^2^	31.26 ± 5.81	27.90 ± 5.81	0.020 ^†^
M protein level, g/dL	4.05 (2.10–6.70)	3.55 (2.10–6.70)	0.967 ^‡^
<3	20 (34.48%)	9 (37.50%)	0.995 ^§^
≥3	38 (65.52%)	15 (62.50%)	
M protein type			
IGG	43 (74.14%)	14 (58.33%)	0.250 ^§^
Other	15 (25.86%)	10 (41.67%)	
sFLC Kappa, mg/L	43.64 (24.50–78.90)	60.03 (29.98–93.44)	0.364 ^‡^
sFLC Lambda, mg/L	33.85 (27.10–61.10)	54.30 (27.20–79.55)	0.149 ^‡^
sFLC Kappa-to-Lambda ratio	1.114 (0.839–1.693)	0.968 (0.683–1.690)	0.558 ^‡^
<1.65	43 (74.14%)	16 (66.67%)	0.678 ^§^
≥1.65	15 (25.86%)	8 (33.33%)	
Risk score			
Low	36 (62.07%)	8 (33.33%)	0.033 ^§^
High	22 (37.93%)	16 (66.67%)	

ASMM, appendicular skeletal muscle mass; COPD, chronic obstructive pulmonary disease. Descriptive statistics are presented using mean ± standard deviation for normally distributed numerical variables, median (25th percentile–75th percentile) for non-normally distributed numerical variables, and count (percentage) for categorical variables. ^†^ Student’s *t*-test; ^‡^ Mann–Whitney U test; ^§^ chi-square test; ^#^ Fisher’s exact test.

**Table 6:** Demographics and clinical characteristics of patients with regard to ASMM index.

	ASMM Index		
Variables	Normal (*n* = 54)	Low (*n* = 28)	*p*
Age	60.98 ± 11.05	65.25 ± 10.38	0.094 ^†^
Sex			
Female	36 (66.67%)	7 (25.00%)	0.001 ^§^
Male	18 (33.33%)	21 (75.00%)	
Comorbidity			
Hypertension	22 (40.74%)	16 (57.14%)	0.238 ^§^
Diabetes mellitus	16 (29.63%)	9 (32.14%)	1.000 ^§^
Ischemic heart disease	11 (20.37%)	7 (25.00%)	0.842 ^§^
Hypothyroidism	5 (9.26%)	1 (3.57%)	0.659 ^#^
COPD	2 (3.70%)	1 (3.57%)	1.000 ^#^
Hyperlipidemia	2 (3.70%)	0 (0.00%)	0.545 ^#^
Body mass index, kg/m^2^	31.16 ± 5.94	28.57 ± 5.78	0.063 ^†^
M protein level, g/dL	4.20 (2.10–7.00)	3.55 (2.00–5.75)	0.428 ^‡^
<3	19 (35.19%)	10 (35.71%)	1.000 ^§^
≥3	35 (64.81%)	18 (64.29%)	
M protein type			
IGG	38 (70.37%)	19 (67.86%)	1.000^§^
Other	16 (29.63%)	9 (32.14%)	
sFLC Kappa, mg/L	44.65 (22.80–87.55)	50.03 (35.53–79.11)	0.577 ^‡^
sFLC Lambda, mg/L	35.05 (26.30–75.00)	47.15 (28.25–71.05)	0.497 ^‡^
sFLC Kappa-to-Lambda ratio	1.054 (0.832–1.510)	1.248 (0.687–1.871)	0.788 ^‡^
<1.65	42 (77.78%)	17 (60.71%)	0.170 ^§^
≥1.65	12 (22.22%)	11 (39.29%)	
Risk score			
Low	32 (59.26%)	12 (42.86%)	0.238 ^§^
High	22 (40.74%)	16 (57.14%)	

ASMM, appendicular skeletal muscle mass; COPD, chronic obstructive pulmonary disease. Descriptive statistics are presented using mean ± standard deviation for normally distributed numerical variables, median (25th percentile–75th percentile) for non-normally distributed numerical variables, and count (percentage) for categorical variables. ^†^ Student’s *t*-test; ^‡^ Mann–Whitney U test; ^§^ chi-square test; ^#^ Fisher’s exact test.

**Table 7:** Demographics and clinical characteristics of patients regarding six-minute walk test.

	Six-Minute Walk Test		
Variables	Normal (*n* = 36)	Low (*n* = 46)	*p*
Age	55.72 ± 10.66	67.70 ± 7.95	<0.001 ^†^
Sex			
Female	19 (52.78%)	24 (52.17%)	1.000 ^§^
Male	17 (47.22%)	22 (47.83%)	
Comorbidity			
Hypertension	13 (36.11%)	25 (54.35%)	0.156 ^§^
Diabetes mellitus	8 (22.22%)	17 (36.96%)	0.231 ^§^
Ischemic heart disease	5 (13.89%)	13 (28.26%)	0.197 ^§^
Hypothyroidism	2 (5.56%)	4 (8.70%)	0.690 ^#^
COPD	0 (0.00%)	3 (6.52%)	0.252 ^#^
Hyperlipidemia	1 (2.78%)	1 (2.17%)	1.000 ^#^
Body mass index, kg/m^2^	30.67 ± 5.31	29.97 ± 6.49	0.603 ^†^
M protein level, g/dL	3.75 (2.20–6.25)	4.20 (2.10–7.00)	0.716 ^‡^
<3	12 (33.33%)	17 (36.96%)	0.914 ^§^
≥3	24 (66.67%)	29 (63.04%)	
M protein type			
IGG	26 (72.22%)	31 (67.39%)	0.818^§^
Other	10 (27.78%)	15 (32.61%)	
sFLC Kappa, mg/L	41.40 (18.82–69.98)	52.15 (33.70–90.67)	0.071 ^‡^
sFLC Lambda, mg/L	32.70 (21.15–50.65)	48.85 (29.70–79.70)	0.014 ^‡^
sFLC Kappa-to-Lambda ratio	1.104 (0.781–2.021)	1.069 (0.739–1.510)	0.695 ^‡^
<1.65	24 (66.67%)	35 (76.09%)	0.487 ^§^
≥1.65	12 (33.33%)	11 (23.91%)	
Risk score			
Low	18 (50.00%)	26 (56.52%)	0.715 ^§^
High	18 (50.00%)	20 (43.48%)	

COPD, chronic obstructive pulmonary disease. Descriptive statistics are presented using mean ± standard deviation for normally distributed numerical variables, median (25th percentile–75th percentile) for non-normally distributed numerical variables, and count (percentage) for categorical variables. ^†^ Student’s *t*-test; ^‡^ Mann–Whitney U test; ^§^ chi-square test; ^#^ Fisher’s exact test.

## Data Availability

Data are available upon reasonable request to the corresponding author.
